# Dislocation of the Astragalus

**Published:** 1874-10-01

**Authors:** 


### DISLOCATION OF THE ASTRAGALUS.

### Case in the Practice of Dr. Isham.

*Reported by Dr. Strout.*

ON Thursday, 13th ult., a man was brought into the office who had been thrown from his wagon by a runaway horse.

Although he was not able to tell how he struck, it was evident that he was precipitated upon his feet, as both heels were torn from his boots.

Upon examination the right foot was found inverted, with the toes depressed, and a well-defined, rounded tumor, in front of the tibio-tarsal articulation, immediately beneath the skin, which proved to be the astragalus, on which could be readily traced its upper non-articulating surface, and also the one which articulates with the scaphoid. An anæsthetic was administered and the leg flexed, so as to relax the tendo achilles, when the bone was pushed back into position with very little force or trouble.

There was also produced, in the other leg, what is commonly designated Pott's fracture.

On visiting the patient next day there was the appearance of a slight slough in the integument, where the bone had pressed forwards, caused by the tension produced.

At the end of a week from the time the accident occurred, the Pott's fracture was dressed in plaster-of-Paris bandage, at which time nearly all the swelling and inflammation had subsided in the other foot, and at the present time (2d inst.), it is progressing favorably.

